# A sensitive bacterial-growth-based test reveals how intestinal *Bacteroides* meet their porphyrin requirement

**DOI:** 10.1186/s12866-015-0616-0

**Published:** 2015-12-29

**Authors:** David Halpern, Alexandra Gruss

**Affiliations:** Micalis Institute, INRA, AgroParisTech, Université Paris-Saclay, 78350 Jouy-en-Josas, France, Jouy en Josas, 78352 France

**Keywords:** Heme auxotroph, Protoporphyrin IX, Metabolites, Germfree, Diagnostics, Microbiota

## Abstract

**Background:**

*Bacteroides* sp. are dominant constituents of the human and animal intestinal microbiota require porphyrins (i.e., protoporphyrin IX or iron-charged heme) for normal growth. The highly stimulatory effect of porphyrins on *Bacteroides* growth lead us to propose their use as a potential determinant of bacterial colonization. However, showing a role for porphryins would require sensitive detection methods that work in complex samples such as feces.

**Results:**

We devised a highly sensitive semi-quantitative porphyrin detection method (detection limit 1-4 ng heme or PPIX) that can be used to assay pure or complex biological samples, based on *Bacteroides* growth stimulation. The test revealed that healthy colonized or non-colonized murine and human hosts provide porphyrins in feces, which stimulate *Bacteroides* growth. In addition, a common microbiota constituent, *Escherichia coli*, is shown to be a porphyrin donor, suggesting a novel basis for intestinal bacterial interactions.

**Conclusions:**

A highly sensitive method to detect porphyrins based on bacterial growth is devised and is functional in complex biological samples. Host feces, independently of their microbiota, and *E. coli*, which are present in the intestine, are shown to be porphryin donors. The role of porphyrins as key bioactive molecules can now be assessed for their impact on *Bacteroides* and other bacterial populations in the gut.

**Electronic supplementary material:**

The online version of this article (doi:10.1186/s12866-015-0616-0) contains supplementary material, which is available to authorized users.

## Background

*Bacteroides* are dominant anaerobes of the human intestinal flora [1]. These bacteria are auxotrophic for protoporphyrin IX (PPIX, which is metal-free) or heme (Fe-charged-PPIX) (collectively called porphyrins; for review; [2, 3]). Heme is the active moiety of numerous molecules, including blood hemoglobin. Heme is also toxic due to its oxidative effects [4–6]. Intestinal heme availability may vary between individuals, for example according to diet and overall health conditions [7], and administered purified heme reportedly increases *Bacteroides* populations in the mouse gut [8]. *Bacteroides* are capable of charging PPIX with Fe, and can thus fulfill their heme requirement when supplied with PPIX [9].

Both heme and its PPIX precursor are found in human feces [10, 11]. In view of *Bacteroides*’ strict porphyrin requirement, we hypothesized that fluctuations in natural porphyrin levels in the healthy host could impact *Bacteroides* intestinal colonization. However, sensitive measurements of heme and/or PPIX in healthy individuals are particularly difficult to obtain: Tests such as Hemoccult, which detect heme, are widely used in primary screenings for occult blood as a sign of colorectal disease [12]. These tests have limited sensitivity due to quenching, and may not detect heme at levels present in individuals deemed as healthy. Likewise, commercially available or published biochemical tests, which measure minute amounts of heme, failed in our experience to detect heme in complex biological samples like feces. Other porphyrin detection tests require multi-step extraction procedures and/or have limited sensitivity (see for example [10, 11]). Porphyrin detection in biological samples, particularly in feces, may be important for understanding how these metabolites might impact the microbiota balance of the host. Yet no test to date is satisfactory for estimating porphyrin levels in feces of healthy individuals.

The quasi-strict heme (and in some cases heme or PPIX) requirement of numerous bacteria (reviewed in [2]) led us to devise a porphyrin detection method, referred to as "Heme-PPIX-Screen", based on growth stimulation of *Bacteroides thetaiotaomicron* (Bt) and *Bacteroides fragilis.* As little as 1-4 ng porphyrins was detected in a sample, reflecting the extremely low amounts needed to stimulate *Bacteroides* growth. Our study using Heme-PPIX-Screen revealed two porphyrin sources for *Bacteroides* that may be used in the host intestine. This study provides a novel analytical tool and new results on the sources and potential impact of essential metabolites, heme and its iron-free precursor PPIX, for *Bacteroides* intestinal colonization.

## Results

### Heme-PPIX-screen setup to detect heme *via* Bt growth stimulation

**Fig. 1:**
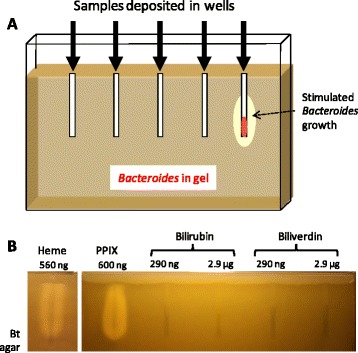
Heme detection setup and specificity of porphyrin detection. **a**. A vertical gel was set up with a 0.8 cm spacer. The gel comprised 0.6 % agarose and growth medium; *Bacteroides* (~10^5^ cfu/ml) was resuspended in medium, which was then poured in the pre-warmed apparatus. Metal prongs (5 cm) were used to form the wells. Samples were loaded in wells, and gels were overlaid with a 5 ml, 1.2 % agar plug. *Bacteroides* growth stimulation was visualized as dense growth around wells after overnight incubation at 37 °C, as schematized for the rightmost well. **b.** Heme and PPIX, but not heme breakdown products stimulated *B. thetaiotaomicron* (Bt) growth. The indicated amounts of the tested products were loaded in wells of the gel setup described in "A". A ~0.5 to 1 cm no-growth-zone from the top of the gel was due to aerobic inhibition of *Bacteroides* growth

A vertical gel setup was designed such that Bt was suspended in porphyrin-free medium in the gel. Samples to be tested are deposited in wells (Fig. 1a; see Methods). Heme (Fe-PPIX), PPIX (no Fe), and heme breakdown products bilirubin and biliverdin were tested (Fig. 1b). Heme and PPIX (>500 ng) both strongly stimulated Bt growth, as expected from *in vitro* studies [9, 13]. In contrast, neither heme degradation product stimulated growth, even at 5-fold higher concentrations. Iron (FeSO_4_ = 0.7 ng and FeCl_3_ = 8 ng) had no effect on Bt growth; no stimulation was seen around empty wells (data not shown). These results indicate that Bt growth stimulation is due to heme and PPIX, but not their breakdown products or iron. Equivalent stimulation was observed when *B. fragilis* was used as the indicator strain (data not shown).

### Porphyrins are detected in complex samples

**Fig. 2:**
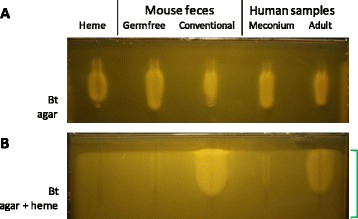
Heme detection in fecal samples. Feces of germ-free and conventional mice, and of newborn and adult humans were tested for their capacity to stimulate *B. thetaiotaomicron* growth in the gel setup. Feces samples resuspended to 100 mg/ml in 0.9 % NaCl were heated to 90 °C for 10 min; 50 μl (5 mg feces) was deposited in each well. Heme (310 ng) was used as control. The deposited samples are as indicated above each well. **a**. The gel contains Bt in M17-glu 0.2 %. **b**. The gel is as in "A", to which we added heme (2.5 μM) to mask the effect of heme supplied by samples. Note that baseline growth of Bt is stimulated by heme throughout the gel (region of the gel indicated at right by the green bracket). The same samples as in "**A**" were loaded. The gel shown was prepared without sugar to lower bubble formation due to CO_2_ production; however, comparable results are obtained in the presence of 0.2 % to 0.5 % glucose. The gels shown are representative of at least 20 germfree and 10 conventional mouse feces, and 5 meconiums and 3 human adult feces samples tested in this way

Feces are highly complex samples comprising digested foods, host-produced products, and usually, a wide range of microbes. Feces were collected from germfree and conventional colonized mice, and newborn and adult humans, and tested for their porphyrin content (Fig. 2a). Remarkably, all fecal samples scored positive in gels. Ground-up and acid-hydrolyzed mouse chow both tested negative, indicating that in these mice, food could be ruled out as a major porphyrin source (data not shown). Moreover, no signal was obtained when Bt was omitted from gels (data not shown), proving that the signal was due to Bt growth, and not penetration of bacteria from the fecal test sample. These results suggested that porphyrins contained in feces stimulated Bt growth.

It was possible that *non*-porphyrin metabolites in feces samples were responsible for the observed Bt growth stimulation. However, as heme or PPIX is a growth prerequisite, we considered it likely that growth stimulation by any other metabolite would occur only if a porphyrin was available. We modified conditions to screen for non-porphyrin growth effectors. We reasoned that if heme was added to the gel in non-limiting supply, other factors stimulating *Bacteroides* growth might be revealed. To test this, we used the same samples and conditions as in Fig. 2a, except that 2 μM heme was added to the Bt-containing gel medium (Fig. 2b). As heme in the gel medium was in sufficient supply, no signal beyond background was observed around the heme-containing well (Fig. 2b leftmost well). Feces from germfree mice and meconium samples were negative in these tests, indicating that a porphyrin is the principal metabolite stimulating Bt growth in germfree (or quasi-germfree) individuals. In contrast, feces from colonized hosts clearly contained *non*-porphyrin metabolites that stimulated Bt growth once heme was supplied in the gel medium (Fig. 2b). Notably, the growth zone diameter surrounding feces from colonized hosts is markedly greater when heme is freely available (compare Fig. 2b, heme in the gel, to Fig. 2a, feces sample as sole porphyrin source), showing that PPIX and/or heme is a *sine qua non* requirement for Bt stimulation by any secondary metabolites. Thus, in Fig. 2a conditions, the amounts of porphryin in samples are growth limiting and determine the diameter of growth stimulation regardless of whether secondary metabolites are present.

These results confirm that fecal samples of healthy individuals contain porphyrins, independently of the nature of ingested food or of the microbiota. The confirmation that porphyrin is the limiting factor for normal *Bacteroides* growth validates the detection method as shown in Fig. 2a.

### Feces samples contain heme

Growth of the Bt indicator strain was activated by both heme and PPIX. To determine whether heme was indeed present in feces samples we used derivatives of *Lactococcus lactis*, a heme auxotroph [2], as indicator strains, in particular a ferrochelatase mutant (*hemH*) that cannot charge PPIX with iron [14]. The *hemH* indicator is thus heme-specific. As expected, growth of wild type *L. lactis*, like Bt, was stimulated by heme or PPIX (Additional file 1: Figure S1a); however, heme, but not PPIX, stimulated growth of the *hemH* mutant (Additional file 1: Figure S1b). Growth of a *cydA* mutant strain, which is defective for heme utilization, was not stimulated by either molecule (Additional file 1: Figure S1c). Three of the four tested feces samples stimulated growth on both wild type and *hemH* indicator strains, indicating that samples contained heme (compare Additional file 1: Figure S1e to S1d and f). Neither heme nor PPIX was detectable in the tested human adult feces sample; however *L. lactis* is a less sensitive indicator than Bt. A weaker signal on *hemH* than on the wild type *L. lactis* indicator suggests that PPIX may also be responsible for part of the detected signals. This result confirms that heme is present in the tested porphyrin-positive feces samples.

### Semi-quantitative estimation of heme and PPIX

**Fig. 3:**
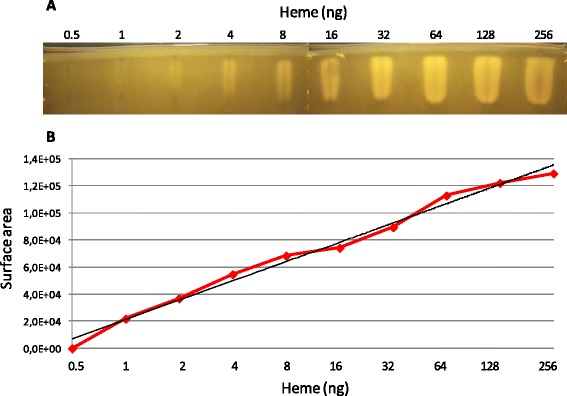
Bt growth stimulation as a function of heme concentrations. **a**. The amount of heme needed to stimulate Bt growth was determined by loading 2-fold dilutions of heme ranging from 256 to 0.5 ng per well. The lowest heme concentration detectably stimulating Bt growth was between 1-4 ng. **b**. Area of growth stimulation was quantified using ImageJ (V1.45 s; Wayne Rasband, National Institute of Health, USA). In black, the logarithmic regression curve of the equation y = 20600ln(x) + 21091, which describes the correlation between the amount of heme and the surface area of stimulated Bt growth, with a correlation coefficient of R^2^ = 0.99

Two-fold dilutions of heme, ranging from 256 ng to 0.5 ng per sample, were loaded in a Bt gel (Fig. 3a). Growth halos were greater with increasing amounts of heme added to wells. The lower threshold of heme-mediated stimulation of Bt growth was approximately 1-4 ng heme per sample. The proportionality between the area of heme-stimulated growth and heme concentration was plotted using ImageJ (Methods), and showed a linear-log relationship (Fig. 3b). In the tested range (1-256 ng heme), two-fold differences in heme concentrations affected the extent of *Bacteroides* growth. We also compared PPIX and heme stimulation in parallel. The same molar amounts of PPIX and heme stimulated Bt growth to similar extents, with about two-fold differences with a given porphyrin concentration (Additional file 1: Figure S2). The Heme-PPIX-Screen assay can thus provide semi-quantitative information on porphyrin availability.

### The Heme-PPIX-Screen assay is highly sensitive for porphyrin detection in complex biological samples

**Fig. 4:**
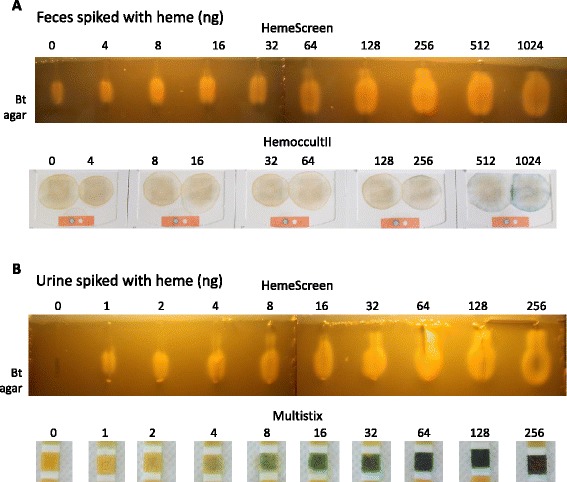
Detection threshold of heme in fecal and urine samples by measuring Bt growth stimulation. **a**. Two-fold heme dilutions were prepared in a feces sample (100 mg resuspended in 1 ml 0.9 % NaCl) from a healthy human adult. Fifty microliters of a feces suspension supernatant (corresponding to 5 mg feces) were loaded per well. Upper, Heme-PPIX-Screen: heme was detected in all samples, including the feces sample without added heme (as in Fig. 2a). An increase in Bt growth stimulation is detected when 64 to 128 ng heme is added to samples, indicating that this amount is distinguishable over the background amount of heme present in a healthy individual. Lower, HemoccultII® test: The same samples as in "Upper" were deposited on the HemoccultII® test and processed as per manufacturer's instructions. The HemoccultII® detection limit of heme in feces was in the range of 512-1024 ng. **b**. Two-fold heme dilutions were prepared in a urine sample from a healthy human donor. Upper, Heme-PPIX-Screen: Heme was detected in all spiked samples, but not in the sample lacking heme. Heme was detected at the lowest amount added, i.e., 1 ng. Lower, Siemens Multistix™ 8SG test: Urine samples as above containing the same heme concentrations were tested with the Siemens Multistix™ 8SG dipstick routinely used to test hematuria and processed as recommended by manufacturer. The lowest heme concentration detected by Siemens Multistix™ 8SG was 4-8 ng. Quantified areas of growth stimulation are shown in Additional file 2: Table S1

We compared the sensitivity of the Heme-PPIX-Screen assay to that of a heme detection test used clinically to measure occult blood in stool and urine. First, preparations of feces from a healthy human adult donor were spiked with increasing amounts of heme, ranging from 4 ng to 1024 ng (Fig. 4a). Using Heme-PPIX-Screen, feces samples without added heme gave a positive signal (Fig. 4a upper, as per Fig. 2). This is expected, as feces of healthy individuals are known to contain porphyrins (usually less than 400 ng heme per 5 mg feces sample [15, 16]). An increased signal was visible starting at 64-128 ng added heme, which was still within the healthy range. Plotting the area of heme-stimulated growth *versus* heme concentration showed a linear log relationship once endogenous porphyrin levels were surpassed by the added heme (as seen on 3 repeats of such gels; Additional file 2: Table S1). We note that compared to the standard with pure heme, the presence of feces lowered test sensitivity by 3- to 4-fold, suggesting that quenching occurs but is minor.

We then tested the same spiked samples as above using the HemoccultII® test (Beckman Coulter), a biochemical heme test based on guaiac oxidation [12] that has been widely used for primary screening of gastro-intestinal pathologies. Samples tested positive starting in the presence of 512-1024 ng heme, as reported (http://www.bestcarelab.com/testsmenu/78.htm) (Fig. 4a lower). In comparison, heme detection by the Heme-PPIX-Screen was about 100 times more sensitive than Hemoccult in our hands. Other heme detection tests have been described, and include multi-step extraction protocols; however, the detection threshold is usually high (e.g., 4 μM in reference [6]). This compares to ~0.1 μM in the Heme-PPIX-Screen assay. These comparisons show that unlike known heme detection tests, only the present assay might be applicable to studies in healthy hosts.

Detection of small amounts of blood in urine may be an indication of pathology in the host, and heme detection is routinely performed in medical laboratories. We spiked urine from a healthy human volunteer with 1 to 256 ng heme per 50 μl urine samples. Using the Heme-PPIX-Screen, heme was detected at 1 ng in a 50 μl urine sample. Compared to the test using the Siemans Multistix 8SG™ dipstick as routinely used in clinical settings, heme was detected at a 4-8-fold greater sensitivity (compare Fig. 4b upper and lower). The heme signal in urine was estimated to be 4 to 5 times lower than that of the standard in pure heme (Additional file 2: Table S1). As normal urine samples are totally negative for porphyrins, samples could be compared for semi-quantification against controls prepared in urine as sample diluent.

These comparisons indicate that if heme or PPIX is present in a complex biological sample, it will be detected using Heme-PPIX-Screen samples without the need for prior extraction or purification, while equivalent quantities would escape detection by other tests. However, as Heme-PPIX-Screen detects both heme and PPIX, a positive signal would more generally indicate that porphyrins are present. Detecting variations in porphyrin concentrations could prove useful in studies of Bt colonization or low-grade inflammation, for which both heme and PPIX play a role [10, 11]. This test would also detect very low amounts of porphyrins in urine, which is associated with urinary tract pathologies [17, 18]. As biological samples result in some signal quenching, heme quantification could be best estimated by preparing control samples in the same biological context as the samples to be tested.

### *E. coli* cross-feeds porphyrins to stimulate Bt growth

**Fig. 5:**
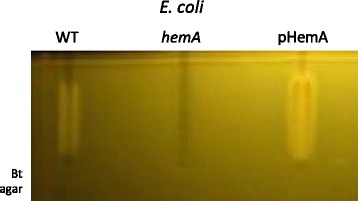
*E. coli* releases porphyrins that stimulate Bt growth. The Heme-PPIX-Screen setup was used to determine whether *E. coli* stimulates Bt growth. *E. coli* WT, ∆*hemA* (heme biosynthesis pathway inactivated), and pHemA (*E. coli* MG1655 containing the pHemA plasmid that induces porphyrin overexpression [25]) were grown overnight. Supernatants of overnight cultures were heated to 90 °C for 10 min and then filter-sterilized, and deposited in the wells

*E. coli* is a common constituent of the human microbiota, and is among the first species to transiently dominate the naïve newborn intestinal flora [19–21]. *E. coli* populations may vary in the host, e.g., after antibiotic treatments [22, 23]. The *Escherichia* synthesize heme, and *E. coli* reportedly secretes heme precursors into the medium [24], leading us to ask whether it could also donate porphyrins to stimulate Bt growth. Wild type (WT) *E. coli* MG1655, a *hemA* mutant derivative that is defective for heme biosynthesis, and MG1655 (pHemA), which overexpresses porphyrins [25], were tested on the Heme-PPIX-Screen setup. The MG1655 and MG1655 (pHemA) strains tested positive as porphyrin donors. However, the *hemA* mutant failed to stimulate Bt growth, confirming that the stimulatory factor is a porphyrin (Fig. 5). Whole cell *E. coli * cultures and filtered culture supernatants were both positive and gave comparable results. These results showed that *E. coli* stimulated Bt growth by cross-feeding porphyrins.

To know about the nature of the secreted porphyrins, we tested *E. coli* supernatants on the *L. lactis* WT and *hemH* indicators. As the signal was not strong enough to be detected by either *L. lactis* strain, we added δ-aminolevulinic acid (100 μg/ml) to *E. coli * cultures, which stimulates the heme biosynthesis pathway [26]. In these conditions, stimulated growth was observed for both WT and *hemH* indicator strains but not the negative control *cydA* strain (Additional file 1: Figure S1g-i). Growth stimulation of the *hemH* indicator strain confirms that heme is among the porphyrins released by *E. coli* in these conditions.

## Discussion

As porphyrins are essential for normal *Bacteroides* growth, these metabolites would expectedly have a growth-limiting role in the intestinal environment [3, 8]. To date, no reliable measure of intestinal porphyrins was available at concentrations present in the healthy host. Here we exploited *Bacteroides* heme auxotrophy to devise the Heme-PPIX-Screen. To our knowledge, bacterial growth was not previously considered as a means to detect porphyrins in samples. The method is simple and highly sensitive, and has the main advantage of functioning in complex samples of essentially any nature, as shown here using several examples. In comparison, biochemical tests measuring heme or PPIX show low sensitivity due to strong quenching, and/or may involve laborious extraction steps. We estimate the detection threshold to be 1-4 ng heme or porphyrin per test sample, based on tests with pure porphyrins, or with heme added to feces, urine, or bacteria. *Bacteroides* are thus highly sensitive indicators due to their extreme dependence on porphyrins for normal growth [27]. The test developed here will allow new questions to be addressed on the factors affecting intestinal microbial equilibrium. The Heme-PPIX-Screen test is well-suited to addressing research questions, in particular those concerning the impact of diet, inflammation, and the microbiota on porphyrin availability in the intestine. Such questions are currently under study in our laboratory using this test.

While Heme-PPIX-Screen is a highly sensitive test, it does have limitations for use. First, samples from a host being treated with antibiotics might inhibit *Bacteroides* growth, in which case the inhibitory zone could override the heme detection zone; this type of event is immediately visible on the gels. Second, the test detects both PPIX (without iron) and heme. While detection of both molecules is medically useful, a refined reporter strain, i.e., which is mutated for its ferrochelatase, could confer specificity for heme. Although *Bacteroides* does not encode a ferrochelatase homolog, it does carry a “deferrochelatase”, as identified in *E. coli* [28, 29]; its inactivation could prove useful for limiting detection exclusively to heme.

Growth-based heme detection could potentially be further simplified: While *Bacteroides* are described as anaerobes, they do tolerate low amounts of oxygen [30], which makes handling straightforward. Use of a more oxygen-tolerant Bt strain could further simplify culture preparation: a recent study revealed the existence of naturally arising *B. fragilis* mutants with higher oxygen tolerance, which acquired mutations in a conserved flavoprotein (BF638R_0963; [31]). Addition of a fluorescence marker to *Bacteroides* [32] might also simplify semi-quantification of growth stimulation.

A main finding using the Heme-PPIX-Screen is that intestinal contents of healthy germfree and colonized mice and humans provide low amounts of heme and PPIX that we propose meet the *Bacteroides* requirement during intestinal colonization. Primocolonization of newborns is a complex process, and bacterial establishment is highly variable [21]. We speculate that variations in porphyrin concentrations among newborns may influence the efficiency of primo-colonization by *Bacteroides*, and possibly other species; this hypothesis is being tested in our laboratory.

A second main finding is that a bacterium of the intestinal microbiota, *E. coli*, is a potential porphyrin donor. Porphyrin cross-feeding may be relevant in vivo, i) in intestinal dysbiosis conditions when *E. coli* populations are high [22, 23], and ii) during primocolonization, when *E. coli* might be among the first populations to colonize the intestine [21, 33]. The test developed here opens perspectives for understanding ecological interactions based on porphyrins.

The importance of the non-heme/PPIX metabolites was uncovered in this study when porphyrins were no longer growth-limiting: feces from colonized adult humans but not newborns, and from conventional but not germfree mice, donated one or more metabolites in addition to porphyrins that stimulated *Bacteroides* growth. By adding heme in the gel (as in Fig. 2b), the *Bacteroides*-based growth stimulation test could thus have an alternative application for identifying bacterial partners that supply *Bacteroides* with metabolites other than porphyrins. Importantly, the Heme-PPIX-Screen is valid even in the presence of secondary metabolites, as porphyrin is the limiting factor that determines the area of *Bacteroides* growth stimulation.

## Conclusions

Heme and PPIX are central metabolites whose presence in the gut i) may lead to shifts in microbiota populations, and ii) is associated with dysbiosis [6, 8]. Porphyrin detection in complex biological samples at normal physiological levels as allowed by our test should be useful for obtaining an understanding of their effects on bacterial equilibrium in the gut.

## Methods

### Strains and growth conditions

The Bt type strain VPI-5482 was from the collection of S.Rabot (INRA, Jouy en Josas, France). *Bacteroides fragilis* ADB77 was generously provided by Michael Malamy (Tufts University, USA;[30]). *E. coli* strain MG1655 (referred to as WT) is a K-12 laboratory strain descendent of a stool culture taken in 1922 (http://www.genome.wisc.edu/resources/strains.htm). The *hemA* mutant is a MG1655 derivative constructed by P1 transduction from the *E. coli* C600 *hemA::km* (kanamycin resistant gene insertion) strain, kindly provided by Cécile Wandersman (Institut Pasteur, France; [34]). The *hemA* overproducer plasmid was kindly sent to us by the Schmidt-Dannert lab (U. Minnesota, USA; [25]). *L. lactis* MG1363 carrying pIL253 (carrying an erythromycin resistance determinant), and MG1363 *hemH* and *cydA* (both erythromycin resistant) strains were from our laboratory collection [14].

Bt cultures were routinely started from 50 μl frozen aliquots prepared as follows: An overnight culture was prepared in M17-glu (glucose 0.5 %) containing heme (10 μM) and cysteine (4 mM); the culture was washed and resuspended in fresh M17-glu containing 15 % glycerol and then aliquoted anaerobically and frozen. For all experiments, Bt cultures were prepared as follows: a frozen aliquot (10 μl) was thawed and resuspended in 10 ml M17-glu (without heme) and grown overnight at 37 °C in an anaerobic chamber (Sheldon Manufacturing Inc., USA) or an anaerobic jar using gas packs (GENbox anaer, BioMerieux). *B. fragilis* cultures were grown as for Bt. *E. coli* strains were grown for heme donation screening in M17-Glu in static conditions at 37 °C. The *E. coli* pHemA strain was grown with 10 μg/ml chloramphenicol. Supernatants of overnight cultures were heated to 90 °C for 10 min and then filter-sterilized, and deposited in the wells. Use of supernatants and heat treatments improved gel resolution, and in the case of the *E. coli* (pHemA) culture was used to inactivate chloramphenicol. *L. lactis* strains were cultured in M17-glu liquid or solid medium with 5 μg/ml erythromycin. Overnight cultures were diluted 1:100 in M17-glu for porphyrin detection tests.

### Additives used in growth assays

Heme, PPIX, biliverdin, and bilirubin were prepared as 20 mM stock solutions as described [35]. Delta-aminolevulinic acid (ALA: Sigma) was added to *E. coli* cultures at 100/μg per mL.

### Commercial heme detection tests

HemoccultII® for detection of heme in feces was purchased from Beckman Coulter (France), and used according to instructions provided with the kit. Multistix™ 8SG (Siemens Healthcare, Germany) for detection of heme in urine was supplied by the INRA-Jouy en Josas campus medical office.

### Setup to screen for heme-containing samples that stimulate *Bacteroides* growth

A vertical gel set-up (Fig. 1a) with 0.8-cm spacers was filled with 0.6 % agar-medium (usually M17-glu unless specified) comprising ~10^5^*Bacteroides* colony forming units (cfu) per ml. The test was named "Heme-PPIX-Screen". In experiments to control for signal specificity, heme (2 μM) was added to *Bacteroides*-containing agar so as to mask growth stimulation due to porphyrins and visualize growth stimulation due to non-porphyrin-metabolites. When heme was added to the gel matrix, sugars were omitted or amounts were reduced to 0.2 % as a means of limiting gas production; note however that residual sugars and possibly other carbon sources in rich medium were sufficient for Bt growth. We designed a "comb" comprising 5 cm-long metal prongs embedded in a hard plastic bar, which was inserted in the gel prior to setting to create deep wells into which test samples were deposited. Bacteria were added to medium just prior to pouring into a pre-warmed gel apparatus. Samples to be tested were deposited in the wells, and gels were then overlaid with a 5 ml, 1.2 % agar plug. As the gel was enclosed by glass plates, bacteria were in oxygen-depleted conditions, so that gels could be handled and incubated in ambient air. Gels were incubated at 37 °C overnight (~16 h), and then examined for Bt growth stimulation and photographed. To correlate growth stimulation with heme concentration, the surface area of growth stimulation was determined using ImageJ (V1.45 s; Wayne Rasband, National Institute of Health, USA), and then plotted against the log of the heme concentration.

### Fecal and urine sample preparation for heme detection

Feces were collected from conventional and germfree mice that were routinely raised in our on-campus animal facilities. Human fecal and urine samples were collected from healthy newborns with parental consent (Professor Claire Poyart program director, Hôpital Cochin, Paris, France), or from healthy human laboratory volunteers. Procedures were carried out in accordance with the Declaration of Helsinki, European Guidelines for the Care and Use of Laboratory Animals, institutional guidelines, and with permission 78-58 of the French Veterinary Services. All samples were used immediately or stored at –80 °C prior to testing.

For heme detection in feces samples, feces were resuspended (100 mg/ml) in 0.9 % NaCl and samples were heated to 90 °C for 10 min to avoid bacterial outgrowth. In some experiments, feces samples were centrifuged and supernatants were used. Similar results were obtained in the absence of heating, but sometimes resulted in bubble formation in gels. For heme detection in urine, a urine sample was used undiluted. In both cases the above-prepared samples were spiked with 2-fold dilutions of heme prior to loading 50 μl samples in wells.

### Bacterial sample preparation for heme detection

Overnight cultures of *E. coli* strains were used directly after heating at 90 °C for ten minutes. Alternatively, culture supernatants were filtered and then heated to 90 °C for 10 min prior to loading 50 μl in wells.

## Additional files

Additional file 1:
**Figure S1.** Heme constitutes part of the porphyrin signal in feces and bacterial samples. *Lactococcus lactis* is a heme auxotroph whose growth is stimulated by heme [14]. *L. lactis* wild type (WT) and ferrochelatase-defective (*hemH*) strains, and a mutant that cannot utilize heme (*cydA*) were used as indicator strains to determine whether heme is produced by test samples. The *hemH* –minus strain lacks the capacity to charge PPIX with iron, and thus growth is not stimulated. Samples are: a-c, PPIX and heme (5 μL of 10 μM stock solutions, as indicated). Note that PPIX does not stimulate growth of the *hemH* indicator; d-f, feces from axenic and conventional (Conven) mice, and from human meconium and adult. Note that in each case, 5 mg of a same sample were deposited on the plates; g-i, filtered supernatants of a WT *E. coli* strain that was grown aerobically overnight in LB supplemented with delta-amino levulinic acid to stimulate heme synthesis [26]. In the lower panels, the weak signals obtained for *E. coli* were Photoshop-amplified by the “automatic levels” option used simultaneously for the three spots. The presence of heme in feces and *E. coli* samples is revealed by zones of stimulated growth in the *hemH* indicator strain (central panels). **Figure S2.** Heme and PPIX stimulate *Bacteroides* growth. A. Identical molar amounts of heme and PPIX were tested for their capacity to stimulate *Bacteroides* growth. *Bacteroides *growth was stimulated when either heme or PPIX was added at 0.1 μM (equivalent to approximately 3 ng). The absence of detectable signal in the central well shows the background level when no sample is added. The gel was prepared in M17 containing 0.5 % glucose into which 7.10^5^ Bacteroides was added per ml agar (see Methods). B. Areas of growth stimulation were quantified using ImageJ (V1.45 s; Wayne Rasband, National Institute of Health, USA). (DOCX 1157 kb) Additional file 2: Table S1. Heme-PPIX-Screen detection of pure heme or in biological samples. (XLSX 20 kb)

## References

[1] Gibson GR, Roberfroid MB (1995). Dietary modulation of the human colonic microbiota: introducing the concept of prebiotics. J Nutr.

[2] Gruss A, Borezee-Durant E, Lechardeur D (2012). Environmental heme utilization by heme-auxotrophic bacteria. Adv Microb Physiol.

[3] Rocha EP, Smith CJ, Cornelis P, Andrews SC (2010). Heme and Iron Metabolism in Bacteroides. Iron uptake and homeostasis in microorganisms.

[4] de Vogel J, van-Eck WB, Sesink AL, Jonker-Termont DS, Kleibeuker J, van der Meer R (2008). Dietary heme injures surface epithelium resulting in hyperproliferation, inhibition of apoptosis and crypt hyperplasia in rat colon. Carcinogenesis.

[5] IJssennagger N, Rijnierse A, de Wit NJ, Boekschoten MV, Dekker J, Schonewille A (2013). Dietary heme induces acute oxidative stress, but delayed cytotoxicity and compensatory hyperproliferation in mouse colon. Carcinogenesis.

[6] Pierre F, Tache S, Petit CR, Van der Meer R, Corpet DE (2003). Meat and cancer: haemoglobin and haemin in a low-calcium diet promote colorectal carcinogenesis at the aberrant crypt stage in rats. Carcinogenesis.

[7] Zuckerman GR, Prakash C, Askin MP, Lewis BS (2000). AGA technical review on the evaluation and management of occult and obscure gastrointestinal bleeding. Gastroenterology.

[8] IJssennagger N, Derrien M, van Doorn GM, Rijnierse A, van den Bogert B, Muller M (2012). Dietary heme alters microbiota and mucosa of mouse colon without functional changes in host-microbe cross-talk. PLoS One.

[9] Sperry JF, Appleman MD, Wilkins TD (1977). Requirement of heme for growth of *Bacteroides fragilis*. Appl Environ Microbiol.

[10] Silva FR, Nabeshima CT, Bellini MH, Schor N, Vieira ND, Courrol LC (2013). Study of protoporphyrin IX elimination by body excreta: a new noninvasive cancer diagnostic method? *J*. Fluorescence.

[11] van den Berg JW, Koole-Lesuis R, Edixhoven-Bosdijk A, Brouwers N (1988). Automating the quantification of heme in feces. Clin Chem..

[12] van Rossum LG, van Rijn AF, Laheij RJ, van Oijen MG, Fockens P, van Krieken HH (2008). Random comparison of guaiac and immunochemical fecal occult blood tests for colorectal cancer in a screening population. Gastroenterol..

[13] Caldwell DR, White DC, Bryant MP, Doetsch RN (1965). Specificity of the heme requirement for growth of *Bacteroides ruminicola*. J Bacteriol..

[14] Duwat P, Sourice S, Cesselin B, Lamberet G, Vido K, Gaudu P (2001). Respiration capacity of the fermenting bacterium *Lactococcus lactis* and its positive effects on growth and survival. J Bacteriol..

[15] Ahlquist DA, McGill DB, Schwartz S, Taylor WF, Ellefson M, Owen RA (1984). HemoQuant, a new quantitative assay for fecal hemoglobin. Comparison with Hemoccult. Annals Int Med.

[16] Vaananen P, Tenhunen R (1988). Rapid immunochemical detection of fecal occult blood by use of a latex-agglutination test. Clin Chem..

[17] Saraf SL, Zhang X, Kanias T, Lash JP, Molokie RE, Oza B (2014). Haemoglobinuria is associated with chronic kidney disease and its progression in patients with sickle cell anaemia. Brit J Haematol..

[18] Wang JP, Qi L, Zheng B, Liu F, Moore MR, Ng JC (2002). Porphyrins as early biomarkers for arsenic exposure in animals and humans. Cell Mol Biol..

[19] Fanaro S, Chierici R, Guerrini P, Vigi V (2003). Intestinal microflora in early infancy: composition and development. Acta Paediatrica.

[20] Orrhage K, Nord CE (1999). Factors controlling the bacterial colonization of the intestine in breastfed infants. Acta Paediatr Suppl.

[21] Palmer C, Bik EM, DiGiulio DB, Relman DA, Brown PO (2007). Development of the human infant intestinal microbiota. PLoS Biol.

[22] Barc MC, Bourlioux F, Rigottier-Gois L, Charrin-Sarnel C, Janoir C, Boureau H (2004). Effect of amoxicillin-clavulanic acid on human fecal flora in a gnotobiotic mouse model assessed with fluorescence hybridization using group-specific 16S rRNA probes in combination with flow cytometry. Antimicrob Agents Chemother..

[23] Barc MC, Charrin-Sarnel C, Rochet V, Bourlioux F, Sandre C, Boureau H (2008). Molecular analysis of the digestive microbiota in a gnotobiotic mouse model during antibiotic treatment: Influence of Saccharomyces boulardii. Anaerobe.

[24] Tatsumi R, Wachi M (2008). TolC-dependent exclusion of porphyrins in *Escherichia coli*. J Bacteriol..

[25] Kwon SJ, de Boer AL, Petri R, Schmidt-Dannert C (2003). High-level production of porphyrins in metabolically engineered *Escherichia coli*: systematic extension of a pathway assembled from overexpressed genes involved in heme biosynthesis. Appl Environ Microbiol..

[26] Avissar YJ, Beale SI (1989). Identification of the enzymatic basis for delta-aminolevulinic acid auxotrophy in a *hemA* mutant of *Escherichia coli*. J Bacteriol..

[27] Baughn AD, Malamy MH (2003). The essential role of fumarate reductase in haem-dependent growth stimulation of *Bacteroides fragilis*. Microbiol.

[28] Letoffe S, Heuck G, Delepelaire P, Lange N, Wandersman C (2009). Bacteria capture iron from heme by keeping tetrapyrrol skeleton intact. Proc Natl Acad Sci U S A.

[29] Turlin E, Heuck G, Simoes Brandao MI, Szili N, Mellin JR, Lange N (2014). Protoporphyrin (PPIX) efflux by the MacAB-TolC pump in *Escherichia coli*. MicrobiologyOpen.

[30] Baughn AD, Malamy MH (2004). The strict anaerobe *Bacteroides fragilis* grows in and benefits from nanomolar concentrations of oxygen. Nature.

[31] Meehan BM, Baughn AD, Gallegos R, Malamy MH (2012). Inactivation of a single gene enables microaerobic growth of the obligate anaerobe *Bacteroides fragilis*. Proc Natl Acad Sci U S A.

[32] Lobo LA, Smith CJ, Rocha ER (2011). Flavin mononucleotide (FMN)-based fluorescent protein (FbFP) as reporter for gene expression in the anaerobe *Bacteroides fragilis*. FEMS Microbiol Lett..

[33] Adlerberth I, Wold AE (2009). Establishment of the gut microbiota in Western infants. Acta Paediatr.

[34] Letoffe S, Debarbieux L, Izadi N, Delepelaire P, Wandersman C (2003). Ligand delivery by haem carrier proteins: the binding of *Serratia marcescens* haemophore to its outer membrane receptor is mediated by two distinct peptide regions. Mol Microbiol.

[35] Fernandez A, Lechardeur D, Derre-Bobillot A, Couve E, Gaudu P, Gruss A (2010). Two coregulated efflux transporters modulate intracellular heme and protoporphyrin IX availability in *Streptococcus agalactiae*. PLoS Pathogens.

