# Supplementary material for: A sensitive bacterial-growth-based test reveals how intestinal Bacteroides meet their porphyrin requirement
Source: BMC Microbiol. 2015 Dec 29;15:282. doi: 10.1186/s12866-015-0616-0 (PMC4696147; doi:10.1186/s12866-015-0616-0)
Supplement: Additional file 1: — Figure S1. Heme constitutes part of the porphyrin signal in feces and bacterial samples. Lactococcus lactis is a heme auxotroph whose growth is stimulated by heme [14]. L. lactis wild type (WT) and ferrochelatase-defective (hemH) strains, and a mutant that cannot utilize heme (cydA) were used as indicator strains to determine whether heme is produced by test samples. The hemH –minus strain lacks the capacity to charge PPIX with iron, and thus growth is not stimulated. Samples are: a-c, PPIX and heme (5 μL of 10 μM stock solutions, as indicated). Note that PPIX does not stimulate growth of the hemH indicator; d-f, feces from axenic and conventional (Conven) mice, and from human meconium and adult. Note that in each case, 5 mg of a same sample were deposited on the plates; g-i, filtered supernatants of a WT E. coli strain that was grown aerobically overnight in LB supplemented with delta-amino levulinic acid to stimulate heme synthesis [26]. In the lower panels, the weak signals obtained for E. coli were Photoshop-amplified by the “automatic levels” option used simultaneously for the three spots. The presence of heme in feces and E. coli samples is revealed by zones of stimulated growth in the hemH indicator strain (central panels). Figure S2. Heme and PPIX stimulate Bacteroides growth. A. Identical molar amounts of heme and PPIX were tested for their capacity to stimulate Bacteroides growth. Bacteroides growth was stimulated when either heme or PPIX was added at 0.1 μM (equivalent to approximately 3 ng). The absence of detectable signal in the central well shows the background level when no sample is added. The gel was prepared in M17 containing 0.5 % glucose into which 7.105 Bacteroides was added per ml agar (see Methods). B. Areas of growth stimulation were quantified using ImageJ (V1.45 s; Wayne Rasband, National Institute of Health, USA). (DOCX 1157 kb) [file 12866_2015_616_MOESM1_ESM.docx]

**Fig. S1 . Heme constitutes part of the porphyrin signal in feces and bacterial samples.** *Lactococcus lactis* is a heme auxotroph whose growth is stimulated by heme [[1](#_ENREF_1)]. *L. lactis* wild type (WT) and ferrochelatase-defective (*hemH*) strains, and a mutant that cannot utilize heme (*cydA*) were used as indicator strains to determine whether heme is produced by test samples. The *hemH* –minus strain lacks the capacity to charge PPIX with iron, and thus growth is not stimulated. Samples are: a-c, PPIX and heme (5 µL of 10 µM stock solutions) . Note that PPIX does not stimulate growth of the *hemH*  indicator; d-f, feces from axenic and conventional (Conven) mice, and from meconium and adult. Note that in each case, 5 mg of a same sample were deposited on the plates; g-i, filtered supernatants of a WT *E. coli*  strain that was grown aerobically overnight in LB supplemented with delta-amino levulinic acid to stimulate heme synthesis [[2](#_ENREF_2)]. In the lower panels, the weak signals obtained for *E. coli* were Photoshop-amplified by the « automatic levels » option used simultaneously for the three spots. The presence of heme in feces and *E. coli* samples is revealed by zones of stimulated growth in the *hemH* indicator strain (central panels).

**Fig. S2 . PPIX and heme stimulate *Bacteroides* growth. A.** Identical molar amounts of heme and PPIX were tested for their capacity to stimulate *Bacteroides* growth. Both heme and PPIX stimulated Bacteroides growth when added at 0.005 nanomoles (equivalent to about 3 ng deposited in the well), and at the higher concentrations. The absence of detectable signal in the central well shows the background level when no sample is added. The gel was prepared in M17 containing 0.5% glucose into which 7.10^5^ Bacteroides was added per ml agar (see Materials and Methods). **B.** Area of growth stimulation was quantified using ImageJ (V1.45s; Wayne Rasband, National Institute of Health, USA) and plotted against the log of the quantity of heme (µm, micromoles) in each well.

**Bibliography**

1. Duwat P, Sourice S, Cesselin B, Lamberet G, Vido K, Gaudu P, Le Loir Y, Violet F, Loubiere P, Gruss A. Respiration capacity of the fermenting bacterium *Lactococcus lactis* and its positive effects on growth and survival. *J Bacteriol.* 2001, 183(15):4509-4516.

2. Avissar YJ, Beale SI. Identification of the enzymatic basis for delta-aminolevulinic acid auxotrophy in a hemA mutant of *Escherichia coli*. *J Bacteriol.* 1989, 171(6):2919-2924.
